# Impact of slow K^+^ currents on spike generation can be described by an adaptive threshold model

**DOI:** 10.1007/s10827-016-0601-0

**Published:** 2016-04-16

**Authors:** Ryota Kobayashi, Katsunori Kitano

**Affiliations:** Principles of Informatics Research Division, National Institute of Informatics, 2-1-2 Hitotsubashi, Chiyoda-ku, Tokyo, Japan; Department of Informatics, SOKENDAI (The Graduate University for Advanced Studies), 2-1-2 Hitotsubashi, Chiyoda-ku, Tokyo, Japan; Department of Human and Computer Intelligence, Ritsumeikan University, 1-1-1 Nojihigashi, Kusatsu, Shiga 525-8577 Japan

**Keywords:** Spike generation mechanism, Slow K^+^ currents, Conductance-based models, Integrate-and-fire models, Model reduction

## Abstract

A neuron that is stimulated by rectangular current injections initially responds with a high firing rate, followed by a decrease in the firing rate. This phenomenon is called spike-frequency adaptation and is usually mediated by slow K^+^ currents, such as the M-type K^+^ current (*I*_*M*_) or the Ca^2+^-activated K^+^ current (*I*_*AHP*_). It is not clear how the detailed biophysical mechanisms regulate spike generation in a cortical neuron. In this study, we investigated the impact of slow K^+^ currents on spike generation mechanism by reducing a detailed conductance-based neuron model. We showed that the detailed model can be reduced to a multi-timescale adaptive threshold model, and derived the formulae that describe the relationship between slow K^+^ current parameters and reduced model parameters. Our analysis of the reduced model suggests that slow K^+^ currents have a differential effect on the noise tolerance in neural coding.

## Introduction

Neuronal adaptation is the change in the responsiveness of a neuron over time. Adaptation may play an important role in the extraction of important information from an ever-changing environment and is the product of several factors, including ion channels, synapses, and network dynamics. In this study, we focus on adaptation at the single neuron level. When a neuron is stimulated by rectangular current injections, it initially responds with a high firing rate, followed by a decrease in the firing rate. This phenomenon is called spike-frequency adaptation and is observed in most pyramidal neurons in various brain areas. The spike-frequency adaptation is usually mediated for by M-type K^+^ current (*I*_*M*_) (Brown and Adams 1980; Adams et al. 1982), Ca^2+^-activated K^+^ current (*I*_*AHP*_) (Brown and Griffith 1983; Madison and Nicoll 1984), Na^+^-activated K^+^ current (Schwindt et al. 1989), or the slow inactivation of Na^+^ current (Fleidervish et al. 1996; Kim and Rieke 2003). In terms of the spike-frequency adaptation generated by slow K^+^ currents, conductance-based models including slow K^+^ channels have been studied. These models can reproduce the electrophysiological properties of a neuron (see Koch 1999 for a review) and provide insights into the underlying biophysical mechanisms.

Studies using the conductance-based models have suggested that the distinct biophysical mechanisms responsible for the spike-frequency adaptation have different impacts on neural coding (Ermentrout et al. 2001; Prescott and Sejnowski 2008). For example, *I*_*M*_ improves spike-timing coding, whereas *I*_*AHP*_ improves spike-rate coding (Prescott and Sejnowski 2008). These results indicate that specific biophysical mechanisms underlying adaptation may impact the coding properties of a neuron. On the other hand, due to the complexity of the detailed models, it remains unclear how the kinetics of slow K^+^ currents influence the spike generation mechanism.

In order to understand the spike generation mechanism, it is essential to reduce the detailed neuron models to simplified models. There have been many attempts to simplify the detailed models (Ermentrout and Kopell 1986; Abbott and Kepler 1990; Destexhe 1997; Kistler et al. 1997; Richardson et al. 2003; Fourcaud-Trocmé et al. 2003 for review Rinzel and Ermentrout 1998; Gerstner and Kistler 2002; Izhikevich 2007). A direct approach to obtain the reduced model is to fit the simplified model to simulated data set generated by the detailed model. This approach has clarified the underlying mechanism of spike generation, such as, integration properties (Kistler et al. 1997; Jolivet et al. 2004), adaptation (Brette and Gerstner 2005), and spike threshold variability (Kobayashi and Shinomoto 2007). However, this approach cannot predict the effect of the detailed model parameters (physiological parameters) on spike generation. Another approach is to develop a mathematical framework to simplify the detailed models. For example, the FitzHugh–Nagumo model and integrate-and-fire models (Gerstner and Kistler 2002) were derived from the Hodgkin–Huxley model (Abbott and Kepler 1990; Richardson et al. 2003).

In this study, we extend the mathematical reduction approach by including the spike history effect that is essential to describe the impact of slow K^+^ currents on spike generation. We show that the detailed conductance-based neuron model can be reduced to a multi-timescale adaptive threshold model (Kobayashi et al. 2009; Yamauchi et al. 2011), and derive the formulae that describe the relationship between the slow K^+^ current parameters and the reduced model parameters. We evaluate the reduced model by predicting spike trains of the detailed model. Finally, we examine the effect of noise on the coding property of a neuron using the reduced model.

## Materials and methods

### Single neuron models

#### Conductance-based model

We analyzed a single-compartment conductance-based model based on a model for the cerebral cortex and thalamic neurons proposed by Pospischil et al. (2008), that was extended to include Ca^2+^-activated K^+^ (AHP) current (Mainen and Sejnowski 1996; Tsubo et al. 2004). The membrane voltage *V* of a neuron is described by the following equation:

1 $$ {C}_m\frac{dV}{dt}=-{I}_L-{I}_{Na}-{I}_{Kd}-{I}_M-{I}_{Ca}-{I}_{AHP}+{I}_{\mathrm{ex}}, $$

where *C*_*m*_ is the membrane capacitance and *I*_ex_ is the external input current. The ionic currents consist of the leak current *I*_*L*_ = *g*_*L*_(*V* − *E*_*L*_), Na^+^ current *I*_*Na*_ = *g*_*Na*_*m*^3^*h*(*V* − *E*_*Na*_), delayed rectifier K^+^ current *I*_*Kd*_ = *g*_*Kd*_*n*^4^(*V* − *E*_*K*_), muscarinic K^+^ current *I*_*M*_ = *g*_*M*_*p*(*V* − *E*_*K*_), Ca^2+^ current *I*_*Ca*_ = *g*_*Ca*_*q*^2^*r*(*V* − *E*_*Ca*_), and AHP current *I*_*AHP*_ = *g*_*AHP*_*s*(*V* − *E*_*K*_), where *g*_*x*_ and *E*_*x*_ are the maximal ionic conductances and the reversal potentials, respectively. The gating variables *w* ∈ {*m*, *h*, *n*, *p*, *q*, *r*, *s*} are described by the Hodgkin − Huxley formalism.

2 $$ \frac{dw}{dt}={\alpha}_w\left(V,\left[{\mathrm{Ca}}^{2+}\right]\right)\left(1-w\right)-{\beta}_w\left(V,\left[{\mathrm{Ca}}^{2+}\right]\right)w, $$

where *α*_*w*_ and *β*_*w*_ are the activation and inactivation functions, respectively (see Table 1 for details), and [Ca^2+^] represents the calcium concentration. The Ca^2+^ concentration is described by (Mainen and Sejnowski 1996; Tsubo et al. 2004)

3 $$ \frac{d\left[{\mathrm{Ca}}^{2+}\right]}{dt}=-{10}^5\cdot \frac{I_{Ca}}{2F}-\frac{\left[{\mathrm{Ca}}^{2+}\right]-{\left[{\mathrm{Ca}}^{2+}\right]}_{\infty }}{\tau_{Ca}}, $$

where *F* = 9.6485 × 10^4^ [C/mol] is the Faraday constant, [Ca^2 +^]_∞_ = 0.05 [*μ*M] is the equilibrium concentration, and *τ*_*Ca*_ is Ca^2+^ time constant. The slow K^+^ current parameters were varied in the ranges *g*_*M*_ ∈ [0.05, 0.4] [mS/cm^2^], *g*_*AHP*_ ∈ [0.05, 0.4] [mS/cm^2^], *τ*_max_ ∈ [0.5, 4] [s], *β*_s_ ∈ [10, 90] [/s], and *τ*_*Ca*_ ∈ [0.1, 0.9] [s]. The remaining parameters are shown in Table 1. This model was solved numerically using the forward Euler integration method with a time step of 0.025 [ms] (Jolivet et al. 2004). We further confirmed that the results were quantitatively the same for a time step of 0.01 [ms].

**Table 1:** Parameters of a detailed conductance-based model

Channel *x*	Gating variables *w*	*α*_*w*_ [/ms]	*β*_*w*_ [/ms]	*g*_*x*_ [mS/cm^2^]	*E*_*x*_ [mV]
Na	*m*	$\frac{-0.32\left(V+45\right)}{e^{-\left(V+45\right)/4}-1}$	$\frac{0.28\left(V+18\right)}{e^{\left(V+18\right)/5}-1}$	50.0	50.0
	*h*	0.128*e* ^− (*V* + 41)/18^	$\frac{4}{1+{e}^{-\left(V+18\right)/5}}$	−	−
K	*n*	$\frac{-0.032\left(V+43\right)}{e^{-\left(V+43\right)/5}-1}$	0.5*e* ^− (*V* + 48)/40^	5.0	−90.0
M	*p*	$\frac{p_{\infty }(V)}{\tau_{\infty }(V)}$	$\frac{1-{p}_{\infty }(V)}{\tau_{\infty }(V)}$	0.1	−90.0
Ca	*q*	$\frac{-0.055\left(V+27\right)}{e^{-\left(V+27\right)/3.8}-1}$	0.94*e* ^− (*V* + 75)/17^	0.001	120
	*r*	0.000457*e* ^− (*V* + 13)/50^	$\frac{0.0065}{1+{e}^{-\left(V+15\right)/28}}$	−	−
AHP	*s*	0.01[Ca^2 +^]	0.02	0.2	−90.0

The ion channel *x*, the gating variable *w*, the activation and inactivation functions *α*_*w*_ and *β*_*w*_, the maximal conductance *g*_*x*_, and the reversal potential *E*_*x*_ are summarized. *α*_*p*_ and *β*_*p*_ are given by the equilibrium value *p*_∞_ and the time constant *τ*_*p*_(*V*), ${p}_{\infty }(V)=\frac{1.0}{1+{e}^{-\frac{V+35}{10}}},\ {\tau}_{\mathrm{p}}(V)={\tau}_{\max }/\left\{3.3{e}^{\left(V+35\right)/20}+{e}^{-\left(V+35\right)/20}\right\}$

The other parameters are *C*_*m*_ = 1.0 [μF/cm^2^], *g*_*L*_ = 0.1 [mS/cm^2^], *τ*_max_ = 1.0 [s] and *E*_*L*_ = − 80 [mV], unless otherwise stated

#### Adaptive threshold models

The potential *u* of a model neuron obeys a linear differential equation,

4 $$ \frac{du}{dt}=-\frac{u}{\tau_m}+\frac{I_{\mathrm{ex}}}{C_m}. $$

where *τ*_*m*_ is the membrane time constant. The neuron generates a spike if the potential *u* reaches the spike threshold *θ*_*u*_(*t*) from below, and the threshold is linearly modulated by spikes (Kobayashi et al. 2009; Yamauchi et al. 2011)

5 $$ \begin{array}{l}\mathrm{If}\;u(t)>{\theta}_u(t)\to\ \mathrm{Emit}\ \mathrm{a}\ \mathrm{spike}\ \mathrm{a}\mathrm{t}\ \mathrm{t}\mathrm{ime}\ t,\\ {}{\theta}_u(t)={\theta}_u^{\infty }+{\displaystyle {\sum}_{k:{t}_k<t}{H}_u\left(t-{t}_k\right),}\ \end{array} $$

where *t*_*k*_ is the k-th spike time, *H*_*u*_(*t*) is the threshold kernel that describes the effect of previous spikes, and the sum is taken up to the most recent spike time. The multi-timescale adaptive threshold (MAT) model (Kobayashi et al. 2009) is a special case of the adaptive threshold model (Eq. (5)). The threshold kernel is given by the sum of exponential functions for each spike in the history,

6 $$ {H}_u(t)=\left\{\begin{array}{ll}0\hfill & \left(t\le 0\right)\hfill \\ {}{\displaystyle {\sum}_{j=1}^L{\alpha}_j{e}^{-t/{\tau}_j}}\hfill & \left(0<t\right)\hfill \end{array}\right., $$

where *L* is the number of exponential functions and *α*_*j*_ and *τ*_*j*_ are the weights and the threshold time constants, respectively.

It is worth noting that the potential *u* of the adaptive threshold model is different from the voltage of the leaky integrate-and-fire (LIF) model (Gerstner and Kistler 2002). The potential does not reset after a spike and continuously integrates the input current, whereas the voltage in the LIF model is reset after each spike.

### Input currents

We used two types of input current *I*_ex_(*t*). The first input is a constant current with a pulse,

7 $$ {I}_{\mathrm{ex}}(t)={I}_c+{q}_c\delta \left(t-{t}_p\right), $$

where *I*_*c*_ [*μ* A/cm^2^] is the strength of the constant current, *q*_*c*_ [nC/cm^2^] and *t*_*p*_ [ms] are the amplitude and timing of the pulse, respectively, and *δ*(*t*) is the Dirac’s delta function. The constant part *I*_*c*_ is tuned to maintain the membrane potential at *V*_*c*_ and the pulse amplitude is set to shift the voltage up to −45 [mV], *q*_*c*_ = *C*_*m*_(−45 − *V*_*c*_). The amplitude should be large enough that the neuron always generates a spike. In all simulations, the neuron was stimulated by the pulse after it achieves the steady state. The second input is an *in vivo-*like current modeled by the Ornstein − Uhlenbeck process (Tuckwell 1988; Kobayashi et al. 2011),

8 $$ \frac{d{I}_{\mathrm{ex}}}{dt}=-\frac{I_{\mathrm{ex}}-\mu }{\tau_{\mathrm{syn}}}+\sqrt{\frac{2{\sigma}^2}{\tau_{\mathrm{syn}}}}\eta (t), $$

where, *μ* and *σ* are the mean and standard deviation (SD) of the input, *τ*_syn_ = 2 [ms] is the synaptic time constant, and *η*(*t*) is the Gaussian white noise with zero mean and unit variance.

### Calculation of the spike threshold

**Fig. 1:**
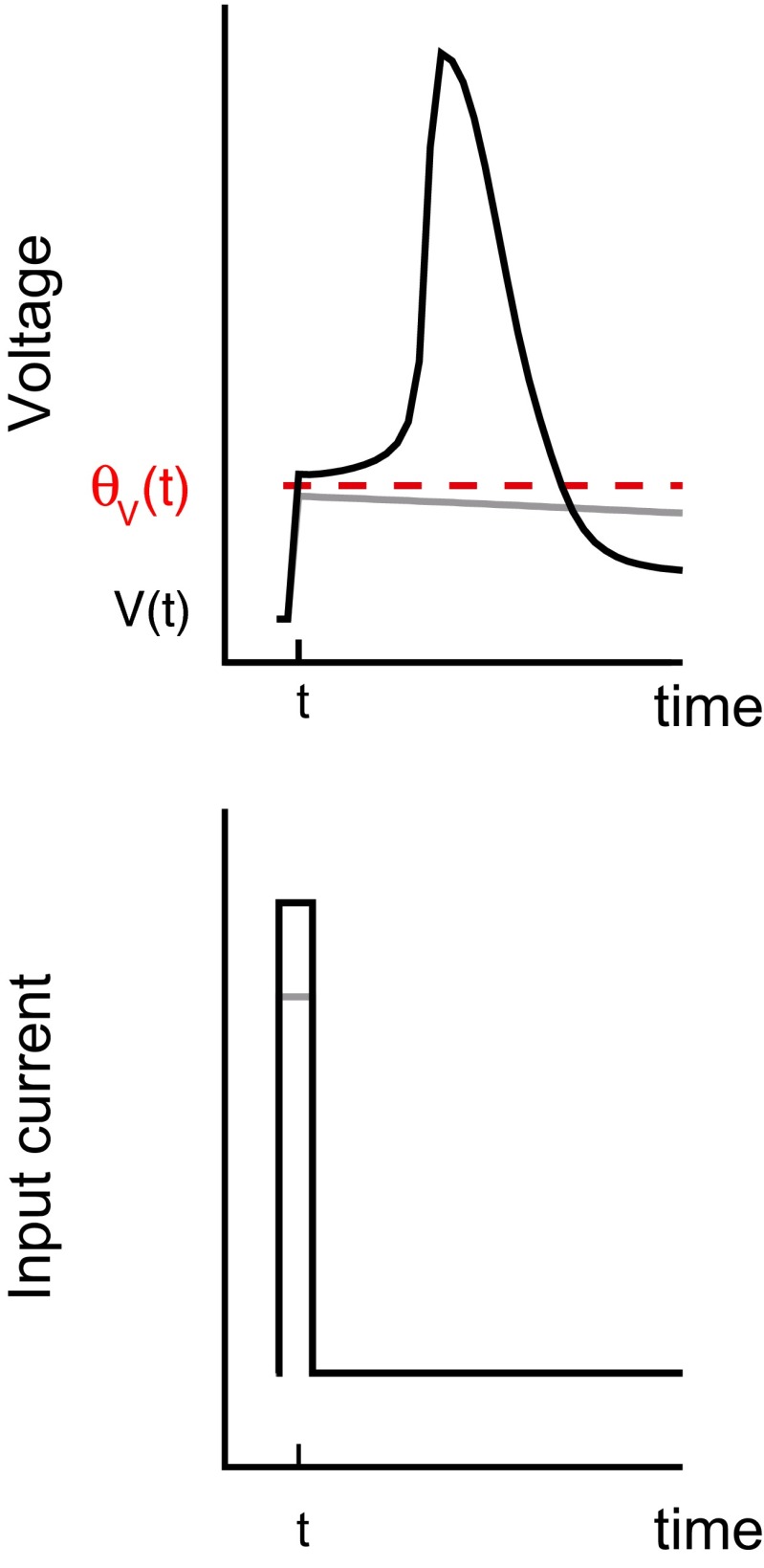
Calculation of spike threshold of a model neuron. The spike threshold *θ*_*V*_(*t*) is defined as the minimal voltage for generating an action potential (*Top*). The minimal voltage is obtained by applying an impulse to the neuron (*Bottom*)

We evaluated the instantaneous spike threshold of the detailed conductance-based model (Eqs. (1), (2), and (3)). To evaluate the spike threshold at time *t*_0_, i.e., *θ*_*V*_(*t*_0_), we stimulate the model neuron with an impulse, *I*_ex_(*t*) = *qδ*(*t* − *t*_0_), and observe whether the model neuron generate a spike or not. The spike threshold is defined as *θ*_*V*_(*t*_0_) = *V*(*t*_0_ − 0) + *q*_min_, where *V*(*t*_0_ − 0) is the voltage immediately before the pulse injection and *q*_min_ is the minimal pulse amplitude for generating a spike (Fig. 1).

The minimal amplitude *q*_min_ can be calculated using the bisection method (Press et al. 2007). Initially, a voltage interval [*a*, *b*] is selected such that *a* (*b*) is lower (higher) than the spike threshold. The initial interval was set as [−80, 0]. Next, we check whether the midpoint *c* = (*a* + *b*)/2 is larger than the spike threshold by observing the voltage for 50 [ms]. If the neuron emits a spike after the voltage shift, *c* is higher than the threshold and the subinterval [*a*, *c*] is selected. Otherwise the subinterval [*c*, *b*] is selected. This procedure is repeated until the interval is sufficiently small (less than 10^− 4^).

### Reduction of a conductance-based neuron model

We have developed a reduction procedure from a detailed conductance-based model (Koch 1999; Izhikevich 2007) to an adaptive threshold model. We start from a conductance-based neuron model described by

9 $$ {C}_m\frac{dV}{dt}=-{\displaystyle {\sum}_{\mathrm{ion}}{I}_{\mathrm{ion}}\left(V,\overrightarrow{w}\right)+{I}_{\mathrm{ex}}(t),} $$

where *I*_ion_ is an ionic current and $\overrightarrow{w}=\left({w}_1,\kern0.5em \cdots, \kern0.5em {w}_d\right)$ is a vector of gating variables. Each gating variable *w*_*i*_ is described by the kinetic equation (2).

The reduction consists of two approximations. First, we assume that a spike threshold *θ*_*V*_(*t*) is written as:

10 $$ {\theta}_V(t)={\theta}_V^{\infty }+{\displaystyle {\sum}_{\mathrm{ion},\ k:{t}_k<t}{h}_{\mathrm{ion}}\left(t-{t}_k\right),} $$

where *θ*_*V*_^∞^ is the spike threshold at the resting state and *h*_ion_ describes the threshold modulation after a spike by an ionic current. If the voltage *V*(*t*) exceeds the threshold *θ*_*V*_(*t*), the neuron generates a spike. In addition, it is assumed that the previous spikes affect the spike threshold linearly. The validity of the assumption was tested by the comparison with the spike threshold of the detailed neuron model (data not shown). The effect of the spike waveform is incorporated into the reset rule. If the voltage exceeds the threshold, we shift the time and the voltage: *t* → *t* + *w*_*sp*_ and *V* → *V* + *δV*, where *w*_*sp*_ is the spike width and *δV* is the voltage change during a spike. Specifically, the spike width *w*_*sp*_ is approximately 2 ∼ 4 [ms] and the voltage change *δV* is −20 ∼ −10 [mV].

Second, we assumed that the ionic currents *I*_ion_ are given by the sum of a spike-triggered ionic current *η*_ion_(*t*) and a leak current in the subthreshold regime (*V* < *θ*_*V*_):

11 $$ {I}_{\mathrm{ion}}\left(V,\overline{w}\right)\approx {\displaystyle {\sum}_{k:{t}_k<t}{\eta}_{\mathrm{ion}}\left(t-{t}_k\right)+{\overline{g}}_{\mathrm{ion}}\left(V-{E}_{\mathrm{ion}}\right),} $$

where ${\overline{g}}_{\mathrm{ion}}$, *E*_ion_ are the average conductance and the reversal potential of an ionic current, respectively. By substituting Eq. (11) into (9), we obtain

12 $$ {C}_m\frac{dV}{dt}=-{g}_{\mathrm{tot}}\left(V-{E}_{\mathrm{tot}}\right)-{\displaystyle {\sum}_{\mathrm{ion},\ k}{\eta}_{\mathrm{ion}}\left(t-{t}_k\right)+{I}_{\mathrm{ex}}(t),} $$

where ${g}_{\mathrm{tot}}={\displaystyle {\sum}_{\mathrm{ion}}{\overline{g}}_{\mathrm{ion}}}$ is the total conductance and ${E}_{\mathrm{tot}}={\displaystyle {\sum}_{\mathrm{ion}}{\overline{g}}_{\mathrm{ion}}{E}_{\mathrm{ion}}/{g}_{\mathrm{tot}}}$ is the effective reversal potential. The formal solution of Eq. (12) can be written as,

13 $$ \begin{array}{l}V(t)={E}_{\mathrm{tot}}-{C}_m^{-1}{\displaystyle {\sum}_{\mathrm{ion},\ k}{\displaystyle {\int}_0^{t-{t}_k}{\eta}_{\mathrm{ion}}\left(t-{t}_k-s\right){e}^{-\frac{s}{\tau_m}}\ ds}}\\ {}\kern10.25em +{C}_m^{-1}{\displaystyle {\int}_0^t{I}_{\mathrm{ex}}\left(t-s\right){e}^{-\frac{s}{\tau_m}}\ ds,}\ \end{array} $$

where *τ*_*m*_ = *C*_*m*_/*g*_tot_ is the effective membrane time constant. The Eq. (13) is a special case of the Spike Response Model (SRM) (Kistler et al. 1997; Gerstner and Kistler 2002; Jolivet et al. 2004). Here, the SRM is used to interpret the effect of the ionic currents on spike generation in the conductance-based model.

Let us consider a new variable *u* that follows a linear equation without resetting after a spike,

14 $$ \frac{du}{dt}=-\frac{u}{\tau_m}+\frac{I_{\mathrm{ex}}}{C_m}, $$

As the solution of Eq. (14) is $u={C}_m^{-1}{\displaystyle {\int}_0^t{I}_{\mathrm{ex}}\left(t-s\right){e}^{-s/{\tau}_m}ds}$, the relationship between the new variable and the voltage is

15 $$ u=V-{E}_{\mathrm{tot}}+{C}_m^{-1}{\displaystyle {\sum}_{\mathrm{ion},\ k}{\displaystyle {\int}_0^{t-{t}_k}{\eta}_{\mathrm{ion}}\left(t-{t}_k-s\right){e}^{-\frac{s}{\tau_m}}ds}}-{\displaystyle {\sum}_k\delta V{e}^{-\frac{t-{t}_k-{w}_{\mathrm{sp}}}{\tau_m}}}, $$

where the last term represents the voltage change during a spike. The spike threshold for *u* can be written as

16 $$ {\theta}_u(t)={\theta}_u^{\infty }+{\displaystyle {\sum}_kH\left(t-{t}_k\right),} $$

where *θ*_*u*_^∞^ = *θ*_*V*_^∞^ − *E*_tot_ and

17 $$ H(t)=-\delta V{e}^{-\frac{t-{w}_{\mathrm{sp}}}{\tau_m}}+{C}_m^{-1}{\displaystyle {\sum}_{\mathrm{ion}}{\displaystyle {\int}_0^t{\eta}_{\mathrm{ion}}\left(t-s\right){e}^{-\frac{s}{\tau_m}}ds}}+{\displaystyle {\sum}_{\mathrm{ion}}{h}_{\mathrm{ion}}(t)}. $$

The effective threshold kernel *H*(*t*) is given by the voltage change during a spike, the spike-triggered ionic currents, and the spike threshold variation after a spike.

It should be noted that only the spike-triggered components of the ionic currents are considered in our framework. However, some of these currents, in particular *I*_*M*_, can be activated at voltages lower than the spike threshold (Prescott and Sejnowski 2008). Thus, the accuracy of the approximation may deteriorate if the voltage fluctuations are large.

### Evaluation of the reduced model

We evaluated the reduced model by predicting the spike train of the detailed model neuron. The predictive performance was evaluated by injecting six fluctuating currents generated by the Ornstein − Uhlenbeck processes (Eq. (8)). The two types of input currents, i.e., the moderately noisy (*σ* = *μ*) input and the highly noisy (*σ* = 2*μ*) input, were examined. For each current type, three values of the mean *μ* were chosen so that the neuron generated spikes with 5, 10, and 20 [Hz]. The input parameters were (*μ, σ*) = (1.98, 1.98), (2.45, 2.45), (3.24, 3.24), (1.33, 2.66), (1.65, 3.30), and (2.22, 4.44) for the neuron with *I*_*M*_ and (*μ, σ*) = (1.84, 1.84), (2.15, 2.15), (2.75, 2.75), (1.28, 2.56), (1.58, 3.16), and (2.10, 4.20) for the neuron with *I*_*AHP*_. Two input–output data sets {*I*(*t*), *V*(*t*)} were obtained by injecting two independent fluctuating currents for 50 [s], which were characterized by the same parameters (*μ*, *σ*, *τ*_*s*_), into the detailed model.

The performance was evaluated based on the coincidence factor Γ (Kistler et al. 1997; Jolivet et al. 2004) defined by

18 $$ \Gamma =\frac{N_c-\left\langle {N}_c\right\rangle }{N_d+{N}_m}\cdot \frac{2}{1-2\nu \Delta}, $$

where *N*_*d*_ and *N*_*m*_ are the number of spikes generated by the detailed model and by the reduced model, *N*_*c*_ is the number of coincidences with precision Δ between the two spike trains, ⟨*N*_*c*_⟩= 2*νN*_*d*_*Δ* is the expected number of coincidences using the Poisson process with the same rate *ν* with which the reduced model generates spikes. The coefficient Γ is 1 only if all the spikes coincided within Δ. A homogeneous Poisson process with the firing rate of the detailed model would yield Γ = 0, which is the chance level. The precision Δ was set to 4 [ms] and the spike time of the detailed model is defined as the time when the voltage crosses 0 [mV].

## Results

### Typical behavior of the detailed conductance-based model

We first observed the behavior of a single-compartment conductance-based model with *I*_Na_, *I*_Kd_, *I*_M_, *I*_Ca_, and *I*_AHP_ (Section 2.1). A rectangular current was injected into the three model neurons, i.e., the neuron with no adaptation (*g*_M_ = *g*_AHP_ = 0 [mS/cm^2^]), the neuron with *I*_M_ (*g*_M_ = 0.1, *g*_AHP_ = 0 [mS/cm^2^]), and the neuron with *I*_AHP_ (*g*_M_ = 0, *g*_AHP_ = 0.2 [mS/cm^2^]).

**Fig. 2:**
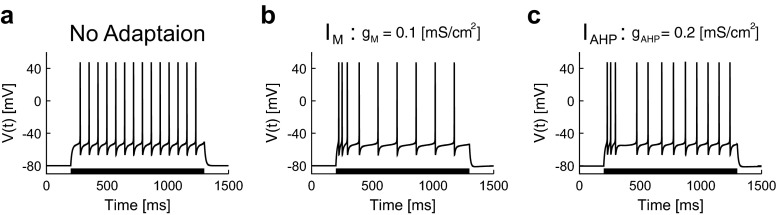
Response of the detailed neuron model to a rectangular current. A rectangular current was injected into the three detailed neurons, i.e., the neuron with no adaptation (**a**: *g*_M_ = *g*_AHP_ = 0 [mS/cm^2^]), the neuron with *I*_M_ (**b**: *g*_M_ = 0.1, *g*_AHP_ = 0 [mS/cm^2^]), and the neuron with *I*_AHP_ (**c**: *g*_M_ = 0, *g*_AHP_ = 0.2 [mS/cm^2^]). The stimulus period (200–1300 [ms]) is indicated as a *black bar*. The input current was 2.5 [μA/cm^2^] (**a**), 3.2 [μA/cm^2^] (**b**), and 3.1 [μA/cm^2^] (**c**). The other parameter values are given in Table 1

 The neuron with no adaption did not exhibit spike-frequency adaptation, i.e., the firing rate does not decrease during the stimulation (Fig. 2a). By contrast, the neuron with *I*_M_ or *I*_AHP_ exhibited spike-frequency adaptation, i.e., the firing rate dropped after the onset of the stimulation (Fig. 2b, c). The firing rate of the neuron with *I*_AHP_ does not decrease gradually, because the neuron has the s-gate for *I*_AHP_. Consistent with previous studies (Benda and Herz 2003; Prescott and Sejnowski 2008), the slow K^+^ currents induced spike-frequency adaptation. Due to the complexity and the nonlinearity of the detailed model, it is not clear how slow K^+^ currents regulate spike generation of a neuron. Thus, we investigated the effects of slow K^+^ currents by mapping the detailed neuron model to a simplified model, and derived a reduced model that clarifies how slow K^+^ currents modulate the effective spike threshold.

### Spike triggered ionic current: *η*_ion_(*t*)

**Fig. 3:**
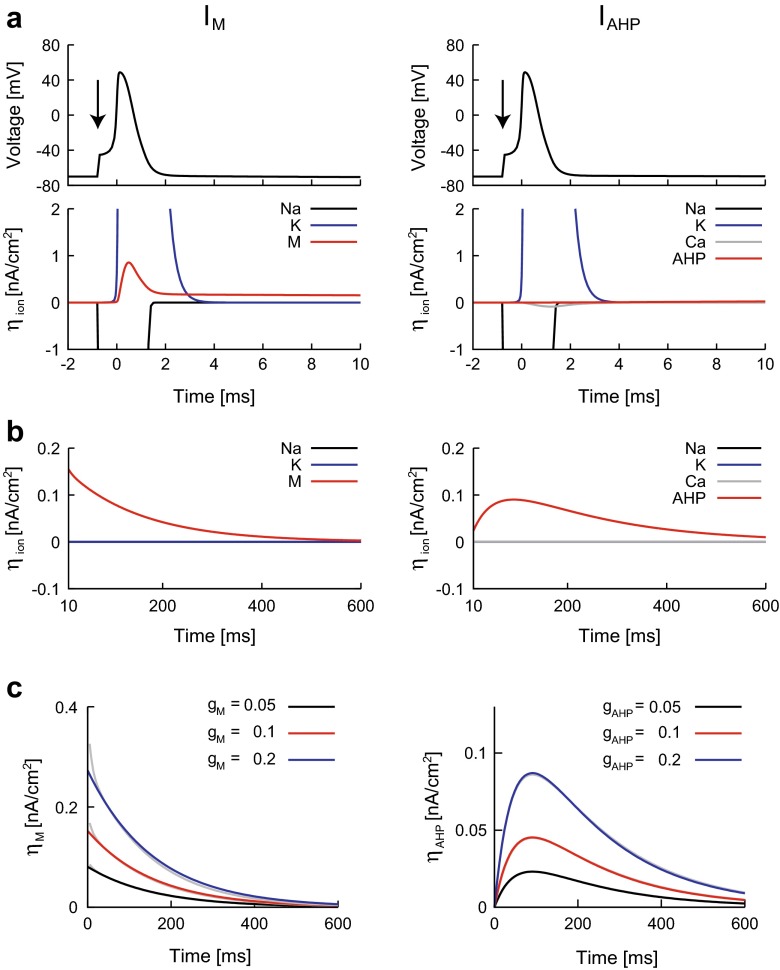
Effects of slow K^+^ currents on the spike-triggered currents. **a**: A constant current with a pulse was injected into the neuron with *I*_M_ (left) and the neuron with *I*_AHP_ (right). *Top and bottom panel* represent the voltage and the spike-triggered currents *η*_ion_(*t*) in the vicinity of a spike, respectively. *Arrows* represent the timing of the pulse injection. **b**: Spike-triggered ionic currents after a spike. **c**: Slow spike-triggered currents *η*_M_(*t*) (left, *gray*) and *η*_AHP_(*t*) (right, *gray*) were compared to the approximate formulae (Eqs. 19, 20) (*black*, *red*, and *blue*). The maximal conductances (*g*_M_, *g*_AHP_) were tested at three levels, i.e., 0.05, 0.1, and 0.2 [mS/cm^2^]. The membrane depolarization was *V*_c_ = − 70 [mV], and the other parameter values were given in Table 1

A constant current with a pulse (Eq. (7)) was injected into the neuron with *I*_M_ and the neuron with *I*_AHP_, and the spike-triggered ionic currents *η*_ion_(*t*) were calculated. Because Na^+^, K^+^, and Ca^2+^ currents vanish within a brief period immediately after the spike (typically 4 [ms] after the spike onset), we focused on analyzing slow K^+^ currents, *I*_M_ and *I*_AHP_ (Fig. 3a, b).

First, we examined the spike-triggered current induced by *I*_M_, *η*_M_(*t*). By replacing an action potential with a rectangular pulse, similar to the approach of Destexhe (1997), the spike-triggered current can be approximated by the exponential function (Appendix A),

19 $$ {\eta}_{\mathrm{M}}(t)\approx {a}_{\mathrm{M}}{e}^{-t/{\tau}_p\left(\overline{v}\right)}, $$

where ${\tau}_p\left(\overline{v}\right)$ is the p-gate time constant and $\overline{v}$ is an average voltage after a spike. The formula (19) is in agreement with *η*_M_(*t*) obtained from the detailed neuron model for various values of *I*_M_ parameters (*g*_M_, *τ*_max_) and membrane depolarization *V*_c_ (Fig. 3c and data not shown for *τ*_max_ and *V*_c_). There is a slight discrepancy in *η*_M_(*t*) between the detailed model and Eq. (19) for small *t*, which may be due to the spike waveform. A more accurate formula can be obtained by incorporating this effect (Appendix A).

Second, we examined the spike-triggered current induced by *I*_AHP_, *η*_AHP_(*t*). By replacing the calcium current with an impulse, the spike-triggered current can be approximated by the sum of two exponentials (Appendix A).

20 $$ {\eta}_{\mathrm{AHP}}(t)\approx {a}_{\mathrm{AHP}}\left({e}^{-t/{\boldsymbol{\tau}}_{\mathrm{Ca}}}-{e}^{-t/{\tilde{\tau}}_s}\right), $$

where *τ*_Ca_ is the Ca^2+^ time constant, ${\tilde{\tau}}_s={\beta}_s^{-1}$ is an approximation of the s-gate time constant, and *β*_*s*_ is the inactivation rate of the s-gate. The formula (20) is also in agreement with *η*_AHP_(*t*) obtained from the detailed neuron model for various values of *I*_AHP_ parameters (*g*_AHP_, *β*_*s*_, and *τ*_Ca_) and the membrane depolarization *V*_c_ (Fig. 3c and data not shown for *β*_*s*_, *τ*_Ca_ and *V*_c_).

### Spike threshold variation by an ionic current: *h*_ion_(*t*)

**Fig. 4:**
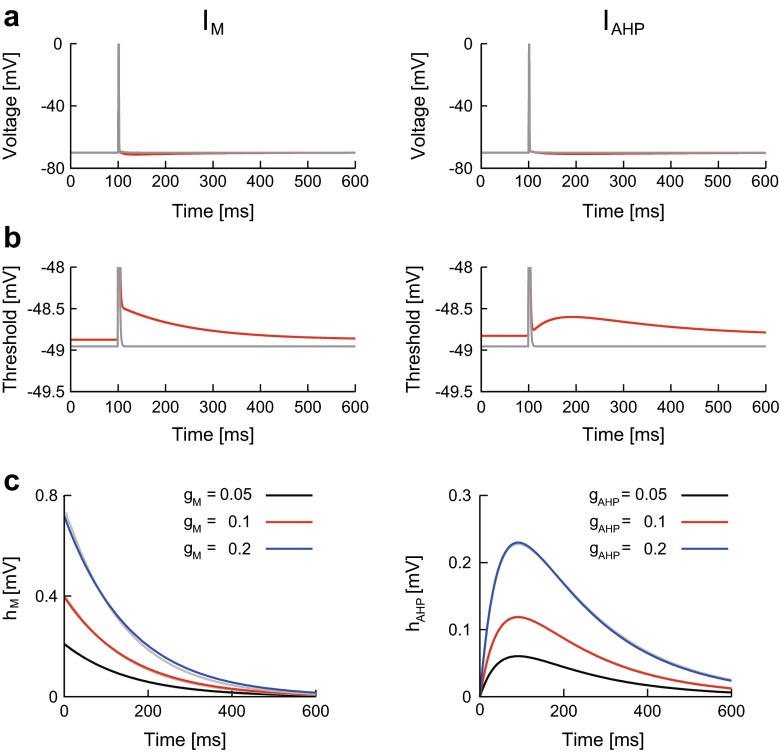
Effects of slow K^+^ currents on the spike threshold. **a**, **b**: The voltage (**a**) and the spike threshold (**b**) of the neuron with *I*_M_ (left, red) and of the neuron with *I*_AHP_ (right, *red*) were compared to the corresponding values for the model neuron with no adaptation (*gray*). **c**: The threshold variations in the detailed model neurons, *h*_M_ (left, *gray*) and *h*_AHP_ (right, *gray*), were compared to the approximate formulae (Eqs. 21, 22) (*black*, *red*, and *blue*). The maximal conductances (*g*_M_, *g*_AHP_) were tested at three levels. Other parameter values were given in Table 1

A constant current with a pulse (Eq. (7)) was injected into the detailed model neurons and the instantaneous spike threshold was calculated (Section 2.3). Again, three neurons were examined, i.e., the neuron with no adaptation, the neuron with *I*_M_, and the neuron with *I*_AHP_. Whereas the spike threshold decays rapidly after a spike in the neuron with no adaptation, it decays slowly in the neuron with *I*_M_ or *I*_AHP_ (Fig. 4b). We can thus conclude that the threshold variation after a spike is mainly caused by the slow K^+^ currents.

The spike threshold variation induced by *I*_M_ was evaluated by comparing the spike threshold in the neuron with *I*_M_ to that with no adaptation. The spike threshold variation is approximately proportional to the spike triggered current *η*_M_(*t*) (Appendix B),

21 $$ {h}_{\mathrm{M}}(t)\approx {b}_{\mathrm{M}}{e}^{-t/{\tau}_p\left(\overline{v}\right)}, $$

where the weight *b*_M_ is proportional to *a*_M_ in Eq. (19). Equation (21) can accurately describe *h*_M_(*t*) for various values of the *I*_M_ parameters (*g*_M_, *τ*_max_) and of the membrane depolarization *V*_c_ (Fig. 4c and data not shown for *τ*_max_ and *V*_c_). Next, the spike threshold variation induced by *I*_AHP_ was evaluated by comparing the threshold in the neuron with *I*_AHP_ to that without *I*_AHP_. The spike threshold variation is approximately proportional to the spike triggered current *η*_AHP_(*t*) (Appendix B),

22 $$ {h}_{\mathrm{AHP}}(t)\approx {b}_{\mathrm{AHP}}\left({e}^{-t/{\tau}_{\mathrm{Ca}}}-{e}^{-t/{\tilde{\tau}}_s}\right), $$

where the weight *b*_AHP_ is proportional to *a*_AHP_ in Eq. (20). Equation (22) can accurately describe *h*_AHP_(*t*) for various values of the *I*_AHP_ parameters (*g*_AHP_, *β*_*s*_, and *τ*_Ca_) and the membrane depolarization *V*_c_ (Fig. 4c and data not shown for *β*_*s*_, *τ*_Ca_ and *V*_c_).

### Reduction of the detailed conductance-based neuron model

The conductance-based neuron model can be reduced to an adaptive threshold model (Section 2.4),

23 $$ \begin{array}{cc}\hfill \frac{du}{dt}=-\frac{u}{\tau_m}+\frac{I_{\mathrm{ex}}}{C_m},\hfill & \hfill \mathrm{If}\;u(t)>{\theta}_u(t)\to\ \mathrm{Emit}\ \mathrm{a}\ \mathrm{spike}\ \mathrm{a}\mathrm{t}\ \mathrm{t}\mathrm{ime}\ t,\hfill \end{array} $$

where *τ*_*m*_ is the membrane time constant and *θ*_*u*_ is the spike threshold for *u* (effective spike threshold) written as

24 $$ {\theta}_u(t)={\theta}_u^{\infty }+{\displaystyle {\sum}_k{H}_u\left(t-{t}_k\right),} $$

*t*_*k*_ is the *k*-th spike time, and *H*_*u*_(*t*) is the effective threshold kernel that describes how the effective spike threshold changes after a spike.

We investigated the effect of the slow K^+^ current parameters on the effective threshold kernel. The threshold kernel *H*_*u*_(*t*) of the neuron with *I*_M_ can be described by the sum of two exponentials,

25 $$ {H}_u(t)\approx {\alpha}_0{e}^{-t/{\tau}_m}+{\alpha}_{\mathrm{M}}{e}^{-t/{\tau}_p\left(\overline{v}\right)}. $$

**Fig. 5:**
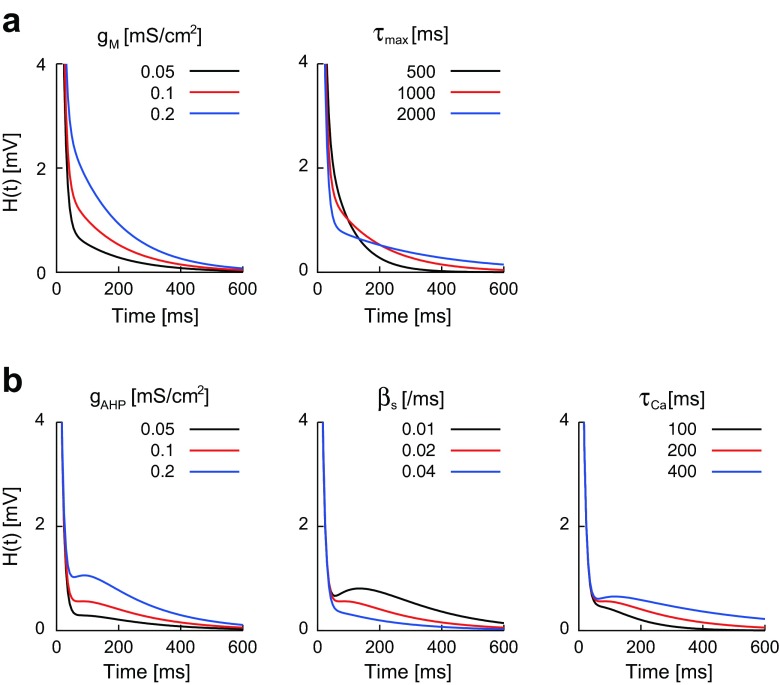
Effective threshold kernel ***H***(***t***) in the detailed neuron model. The effective threshold kernel was calculated from the detailed neuron model with *I*_M_ (**a**) and from the neuron with *I*_AHP_ (**b**). Each parameter (**a**: *g*_M_ and *τ*_max_; **b**: *g*_AHP_, *β*_*s*_, and *τ*_Ca_) was tested at three levels. Other parameter values were given in Table 1

**Fig. 6:**
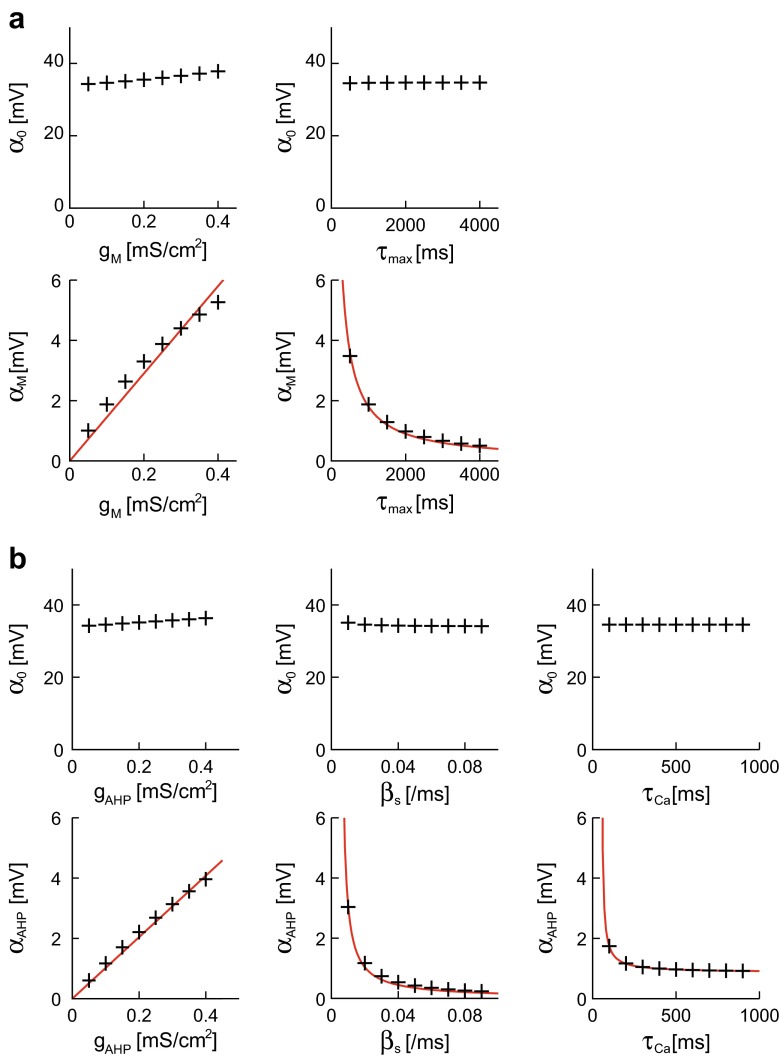
Influence of the slow K^+^ parameters on the threshold kernel. **a**: Scatter plot of the *I*_M_ parameters (*g*_M_, *τ*_max_) in the detailed neuron model vs. the weights of the threshold kernel (*α*_0_, *α*_M_) in the reduced model. **b**: Scatter plot of the *I*_AHP_ parameters (*g*_AHP_, *β*_*s*_, and *τ*_Ca_) in the detailed neuron model vs. the weights (*α*_0_, *α*_AHP_) in the reduced model. Fitted results from the detailed model (crosses) were compared with the approximate formula (Eq. 26, 28, *red lines*)

The threshold kernel is always a monotonically decreasing function in the neuron with *I*_M_ (Fig. 5a). We can derive a formula that clarifies the relationship between the slow weight *α*_M_ and *I*_M_ parameters (Appendix C),

26 $$ {\alpha}_{\mathrm{M}}\propto {g}_{\mathrm{M}}\left(\overline{v}-{E}_K\right)\delta p/{\tau}_{\max }, $$

where *δp* is the changes in the p-gate variable during a spike. As predicted by Eq. (26), the slow weight *α*_M_ increases as *g*_M_ increases, and decreases as *τ*_max_ increases (Fig. 6a). Numerical results indicate that *I*_M_ parameters does not affect on the fast weight *α*_0_ significantly (Fig. 6a).

The threshold kernel *H*_*u*_(*t*) of the neuron with *I*_AHP_ is described by the sum of three exponentials,

27 $$ {H}_u(t)\approx {\alpha}_0{e}^{-t/{\tau}_m}+{\alpha}_{\mathrm{AHP}}\left({e}^{-t/{\tau}_{\mathrm{Ca}}}-{e}^{-t/{\tilde{\tau}}_s}\right). $$

Interestingly, the threshold kernel can be a non-monotonic function in the neuron with *I*_AHP_, and a hump was observed in *H*_*u*_(*t*) (Fig. 5b). We can also derive a formula that clarifies the relation between the slow weight *α*_AHP_ and *I*_AHP_ parameters (Appendix C),

28 $$ {\alpha}_{\mathrm{AHP}}\propto {g}_{\mathrm{AHP}}\left(\overline{v}-{E}_K\right)\delta \mathrm{C}\mathrm{a}\frac{\tau_{\mathrm{Ca}}{\tilde{\tau}}_s}{\tau_{\mathrm{Ca}}-{\tilde{\tau}}_s}, $$

where *δ*Ca is the changes in Ca^2+^ concentration during a spike. As predicted by Eq. (28), the slow weight *α*_AHP_ increases as *g*_AHP_ increases, and decreases as *β*_*s*_ or *τ*_Ca_ increases (Fig. 6b). Numerical results indicate that *I*_AHP_ parameters does not affect on the fast weight *α*_0_ significantly (Fig. 6b).

### Validation of the reduced model

**Fig. 7:**
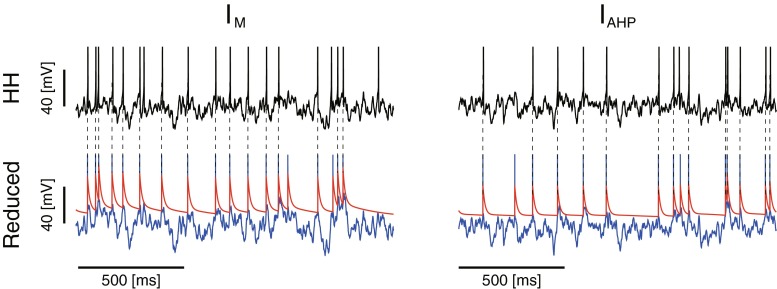
The reduced model can predict the spike timing of the detailed model. Top: Voltage traces of the detailed neuron model with *I*_M_ (*left*) and that with *I*_AHP_ (*right*). Bottom: Spike timing prediction by the reduced model. The coincidence spikes within 4 [ms] were connected by *dotted lines* and the predictive score Γ were 0.85 (*left*) and 0.87 (*right*). *Blue* and *red* represent the potential *u* and threshold *θ*_*u*_, respectively. The parameters were *g*_M_ = 0.2 [mS/cm^2^], *μ* = 2.45 [V/s], and *σ* = 2.45 [mV/√ms] (*left*) and *g*_AHP_ = 0.2 [mS/cm^2^], *μ* = 2.4 [V/s], and *σ* = 2.4 [mV/√ms] (*right*)

We evaluated the reduced model (Eqs. (23), (24), (25), and (27)) by predicting spike trains of the detailed neuron model using the reduced model. Two sets of input–output data {*I*(*t*), *V*(*t*)} (training data and test data) were generated by injecting fluctuating currents (Eq. (8)) to the detailed neuron model for 50 [s]. The reduced model parameters $\left\{{C}_m,{\theta}_u^{\infty },{\alpha}_0,{\tau}_m;{\alpha}_{\mathrm{M}},\kern0.5em {\tau}_p(v);{\alpha}_{\mathrm{AHP}},{\tau}_{\mathrm{Ca}},{\tilde{\tau}}_s\Big\}\right\}$ were tuned from an input–output data set (training data). The membrane capacitance and *I*_AHP_ time constants were adapted from the detailed model, i.e., *C*_*m*_ = 1.0 [nF/cm^2^], *τ*_Ca_ = 200 [ms], and ${\tilde{\tau}}_s=50\left[\mathrm{ms}\right]$, and the membrane time constant was inferred from the leak conductance *τ*_*m*_ = 10 [ms]. The *p*-gate time constant was approximated by its average, ${\tau}_p(v)\approx {\tau}_p\left(\overline{v}\right)$, where $\overline{v}$ is the average voltage. The threshold parameters {*θ*_*u*_^∞^, *α*_0_, *α*_M_} for the neuron with *I*_M_ and {*θ*_*u*_^∞^, *α*_0_, *α*_AHP_} for the neuron with *I*_AHP_, were determined by maximizing the coincidence factor *Γ* (Section 2.5) using the simplex downhill method (Kobayashi et al. 2009). Then, the predictive performance was evaluated by calculating the coincidence factor from the other data set (test data) that was not used for parameter optimization. We found that the reduced model can accurately predict spike trains of the detailed model (Fig. 7). The predictive performance *Γ* for the input currents was 0.854 ± 0.01 (means ± standard errors, unless stated otherwise) for the neuron with *I*_M_, and 0.903 ± 0.01 for the neuron with *I*_AHP_, and the results are summarized in Table 2. The threshold parameters were *θ*_*u*_^∞^ = 30.7 [mV], *α*_0_ = 35.5 [mV], and *α*_M_ = 4.1 [mV] for the neuron with *I*_M_ and *θ*_*u*_^∞^ = 30.7 [mV], *α*_0_ = 32.9 [mV], and *α*_AHP_ = 2.1 [mV] for the neuron with *I*_AHP_.

**Table 2:** Accuracy of spike prediction using the reduced model

Current	Firing rate [Hz]	Γ (with *I*_M_)	Γ (with *I*_AHP_)
M	5	0.823	0.884
M	10	0.805	0.916
M	20	0.854	0.907
H	5	0.886	0.919
H	10	0.894	0.901
H	20	0.862	0.892

The performance of spike prediction for the detailed neuron models using the reduced model is summarized. Each neuron was injected with six fluctuating input currents. Current “M” denotes moderately noisy input (*σ* = *μ*) and current “H” denotes highly noisy input (*σ* = 2*μ*)

### Coding property of the reduced model

We analyzed the impact of slow K^+^ currents on the coding property of a neuron using the reduced model. Here, we focused on the effect of the input noise on firing irregularity. First, we considered that the reduced model neuron (Eqs. (23) and (24)) is stimulated by a constant current, *I*_ex_(*t*) = *I*_0_. An asymptotic periodic solution with period *T* is written as

29 $$ u(t) = {I}_0{\tau}_m, {\theta}_u(t)={\theta}_u^{\infty }+{\eta}_T^{\infty}\left(t-{t}_f\right). $$

where *t*_*f*_ is the most recent spike time, and *η*_*T*_^∞^(*t*) describes the threshold variation between the spikes; *η*_*T*_^∞^(*t*) for the neuron with *I*_M_ is

30 $$ {\eta}_T^{\infty }(t)={\alpha}_0\frac{e^{-t/{\tau}_m}}{1-{e}^{-T/{\tau}_m}}+{\alpha}_M\frac{e^{-t/{\tau}_p\left(\overline{v}\right)}}{1-{e}^{-T/{\tau}_p\left(\overline{v}\right)}}, $$

and *η*_*T*_^∞^(*t*) for the neuron with *I*_AHP_ is

31 $$ {\eta}_T^{\infty }(t)={\alpha}_0\frac{e^{-t/{\tau}_m}}{1-{e}^{-T/{\tau}_m}}+{\alpha}_{AHP}\left(\frac{e^{-t/{\tau}_{\mathrm{Ca}}}}{1-{e}^{-T/{\tau}_{\mathrm{Ca}}}}-\frac{e^{-t/{\tilde{\tau}}_s}}{1-{e}^{-T/{\tilde{\tau}}_s}}\right)\ . $$

The spike condition at the next spike, *t* = *t*_*f*_ + *T*, leads to

32 $$ {\theta}_u\left({t}_f+T\right)={\theta}_u^{\infty }+{\eta}_T^{\infty }(T)={I}_0{\tau}_m. $$

**Fig. 8:**
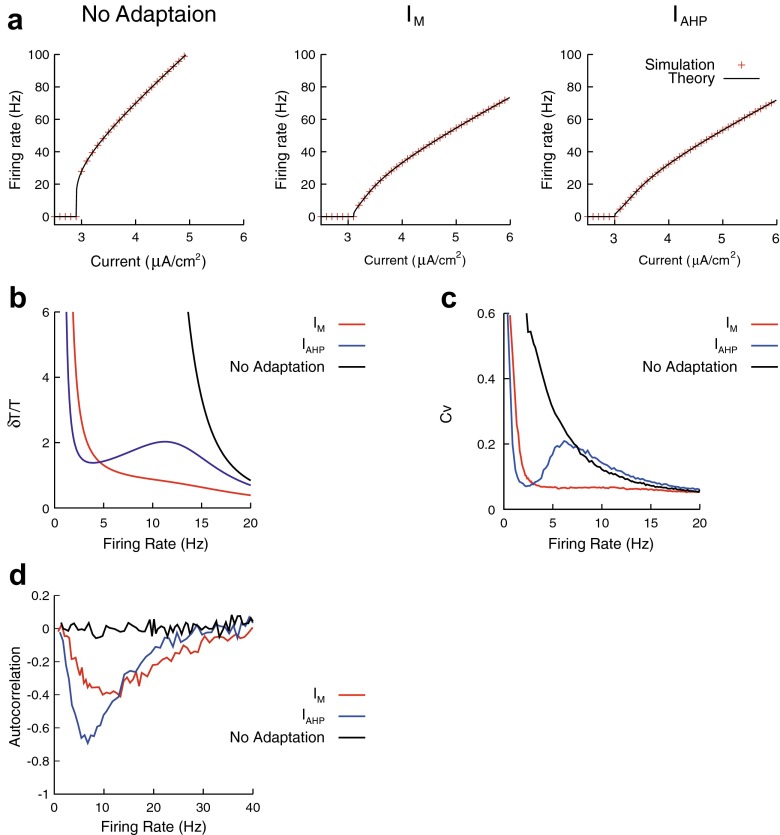
Effect of the input noise on the coding property of a neuron. **a**: *f-I* curve of the reduced neurons. Analytical result (Eq. 34, *black line*) was compared with the simulation result (*red*). The reduced model parameters were *θ*_*u*_^∞^ = 29 [mV], *α*_0_ = 35 [mV], and *τ*_*m*_ = 10 [ms] for the neuron without adaptation, *θ*_*u*_^∞^ = 31 [mV], *α*_0_ = 36 [mV], *α*_M_ = 1.6 [mV], *τ*_*m*_ = 10 [ms], and ${\tau}_p\left(\overline{v}\right)=150$ [ms] for the neuron with *I*_M_ and *θ*_*u*_^∞^ = 30 [mV], *α*_0_ = 34 [mV], *α*_AHP_ = 2.6 [mV], *τ*_*m*_ = 10 [ms], *τ*_*Ca*_ = 200 [ms], and *τ*_*s*_ = 100 [ms] for the neuron with *I*_AHP_. **b**: Effect of the slow K^+^ currents on spiking irregularity (Analytical result, Eq. 35 with *δu* = 1). **c**: Effect of the slow K^+^ currents on Cv of ISIs (Simulated result). Fluctuate currents (Eq. 8) were injected to the detailed model neurons. The input mean *μ* was changed to control the firing rate, whereas the input variance *σ* was fixed: *σ* = 0.04 [mV/√ms]. The neuron parameters were *g*_M_ = 0.1 [mS/cm^2^] for the neuron with *I*_M_ and *g*_AHP_ = 0.1 [mS/cm^2^], *β*_*s*_ = 0.01 [/ms] for the neuron with *I*_AHP_, and other parameter were given in Table 1. **d**: Effect of the slow K^+^ currents on autocorrelation. The autocorrelation was calculated from spike trains generated from the reduced model. The parameters were same as **a**

We can analytically evaluate the firing rate *f* = *T*^− 1^ by solving Eq. (32), and the analytical results are in agreement with *f-I* curves calculated from simulated spike trains (Fig. 8a). The *f-I* curve of the neuron without adaptation (*g*_*M*_ = *g*_*AHP*_ = 0) can be explicitly written as

33 $$ f={\tau}_m^{-1}{ \log}^{-1}\left(1+\frac{\alpha_0}{I_0{\tau}_m-{\theta}_u^{\infty }}\right), $$

which is similar to the *f-I* curve of the LIF neuron. Note that the response of the reduced model with *I*_M_ to the constant current (Eqs. (29) and (30)) is equivalent to the response of the time-dependent threshold model (Tuckwell 1978; Lindner and Longtin 2005; Tamborrino 2016).

Next, we examined the effect of the input noise on spiking irregularity. We have not been able to derive a full analytical result for this effect; however, it is possible to predict the effect of the input noise with the following argument. Let us consider a situation in which a neuron is stimulated by the constant current before the *N*-th spike (*N* ≫ 1) and stimulated by the constant current with small noise after the *N*-th spike. We can evaluate how the input noise changes the timing of the subsequent spike. At the *(N + 1)*-th spike time, the threshold should cross the potential

34 $$ {\theta}_u\left({t}_N+T+\delta T\right)={I}_0{\tau}_m+\delta u, $$

where *t*_*N*_ is the *N*-th spike time, *δT* and *δu* are perturbations due to the small noise. By Taylor-expanding *θ*_*u*_ assuming that *δT* is small, we obtain

35 $$ \delta T\approx \raisebox{1ex}{$\delta u$}\!\left/ \!\raisebox{-1ex}{$\frac{d{\eta}_T^{\infty }}{dt}(T)$}\right.. $$

Equation (35) indicates that slow K^+^ currents improve the robustness against noise in a different manner (Fig. 8b), i.e., *I*_M_ suppresses the spike interval dispersion *δT*/T for a broad firing range, whereas *I*_AHP_ suppress the dispersion only at a low firing range (~3 [Hz]). The dispersion *δT*/*T* is not identical to the coefficient of variation (Cv) of interspike intervals (ISIs), however it has a close relation to Cv. We found that this differential effect was also observed in Cv of the detailed model with slow K^+^ currents (Fig. 8c). Finally, we examined how the slow K^+^ currents modulate autocorrelation of a spike train that was defined as *ρ*_1_ = 〈*ISI*_*i*_*ISI*_*i* + 1_ − 〈*ISI*_*i*_〉^2^〉/〈*ISI*_*i*_^2^ − 〈*ISI*_*i*_〉^2^〉, where *ISI*_*i*_ is the *i*-th ISI and 〈 … 〉 is the averaging over index *i*. The autocorrelation quantifies how often a long ISI is followed by a short ISI and vice versa. The neuron model with the slow K^+^ currents can reproduce the negative ISI correlation, which was commonly observed in sensory periphery and cortical neurons (Farkhooi et al. 2009). As shown in Fig. 8d, the effect of *I*_AHP_ on the autocorrelation is stronger than that of *I*_M_ in the low firing rate regime (<15 [Hz]), whereas the effect of *I*_AHP_ is similar to that of *I*_M_ in the high firing rate regime (>15 [Hz]). A previous work (Chacron et al. 2001) showed that the negative ISI correlation can improve the capacity for encoding time-varying stimulus. Our result implies that the slow K^+^ currents improve the encoding of time-varying stimulus in a different way.

## Discussion

We have shown that the detailed conductance-based neuron model with slow K^+^ currents (*I*_M_ and *I*_AHP_) can be reduced to an adaptive threshold model. The reduced model is written as

36 $$ \begin{array}{l}\begin{array}{cc}\hfill \frac{du}{dt}=-\frac{u}{\tau_m}+\frac{I_{\mathrm{ex}}}{C_m},\hfill & \hfill \mathrm{If}\;u(t)>{\theta}_u(t)\to\ \mathrm{Emits}\ \mathrm{a}\ \mathrm{spike}\ \mathrm{a}\mathrm{t}\ \mathrm{t}\mathrm{ime}\ t,\hfill \end{array}\\ {}{\theta}_u(t)={\theta}_u^{\infty }+{\displaystyle {\sum}_k{H}_u\left(t-{t}_k\right),}\\ {}{H}_u(t)={\alpha}_0{e}^{-t/{\tau}_m}+{\alpha}_{\mathrm{M}}{e}^{-t/{\tau}_p\left(\overline{v}\right)}+{\alpha}_{\mathrm{AHP}}\left({e}^{-t/{\tau}_{\mathrm{Ca}}}-{e}^{-t/{\tilde{\tau}}_s}\right),\end{array} $$

where *θ*_*u*_ is the spike threshold for *u*, and *H*_*u*_(*t*) is the threshold kernel. We have also derived formulae that describe the relationship between slow K^+^ current parameters and reduced model parameters (Eqs. (26) and (28)), which provide a physiological interpretation of the reduced model. The reduced model can accurately predict spike trains of the detailed model (Fig. 7). Our analysis of the reduced model revealed that slow K^+^ currents have differential effects on noise tolerance of a neuron, i.e., *I*_M_ suppresses firing irregularity regardless of the firing rate, whereas *I*_AHP_ suppresses firing irregularity only at a low firing range (Fig. 8b, c). The slow K^+^ currents induce negative interspike interval correlations, and the effect of *I*_AHP_ is stronger than that of *I*_M_ in the low firing regime (Fig. 8d).

### Mapping a detailed conductance-based neuron model to a simplified model

As noted in the Introduction, one approach of obtaining a reduced model is to develop a mathematical framework from detailed neuron models to simplified models. This approach has clarified the relationship between these models. For example, the FitzHugh–Nagumo model was derived from the Hodgkin–Huxley model by assuming that Na^+^ activation (*m*) is instantaneous and that Na^+^ inactivation (*h*) and K^+^ activation (*n*) change with a similar time constant (Abbott and Kepler 1990; Rinzel and Ermentrout 1998). A generalized integrate-and-fire model can also be derived from the Hodgkin–Huxley model by linearization (Destexhe 1997; Koch 1999; Richardson et al. 2003).

In this study, we have extended the linearization approach by including the spike history effect, which is essential for describing the effect of slow K^+^ currents on spike generation. The linearized model is a simple linear equation with the effective threshold *θ*_*u*_(*t*) (Eq. (36)) that incorporates the effect of ionic currents and spike threshold variation on neuronal excitability. We have shown that the effective threshold obtained from the detailed model with slow K^+^ current can be approximated by a modified multi-timescale adaptive threshold (MAT) model (Kobayashi et al. 2009).

### Reduced neuron model

Spike-frequency adaptation can be described by simplified models with adaptation, which is modeled by adaptive current (Liu and Wang 2001; Brette and Gerstner 2005; Izhikevich 2007) or adaptive threshold (Chacron et al. 2000, 2007; Liu and Wang 2001; Jolivet et al. 2004, 2006, 2008). The adaptive threshold models can reproduce the interspike interval statistics (Chacron et al. 2000), *f-I* curve (Rauch et al. 2003; Kobayashi 2009), and spike timings (Jolivet et al. 2006, 2008) of a neuron recorded in experiments. On the other hand, the adaptive threshold model was criticized because, unlike the adaptive current model, it cannot reproduce the lateral shift of *f*-*I* curves observed in experiments (Benda et al. 2010). Note that the derived MAT model (Eq. (36)) incorporates both effects, i.e., the effect of the adaptive current and threshold. This fact can explain the success of the MAT model in accurately predicting spike times (Kobayashi et al. 2009; Yamauchi et al. 2011).

The derived model has two advantages. First, the model is essentially linear; the linearity makes mathematical analysis tractable. Indeed, it is possible to examine the effect of noise on firing irregularity, which can predict a qualitative behavior of the detailed model (Fig. 8). In addition, the linearity enables us to efficiently simulate a network of neurons by the exact sub-threshold integration (Morrison et al. 2007; Yamauchi et al. 2011). Second, the reduced model offers a clear relationship between the slow K^+^ parameters and reduced model parameters (Eqs. (26) and (28)). This relationship is important because it enables us to analyze the effect of slow K^+^ currents using the reduced model.

### Spike threshold variation in experiments

Conventionally, it was considered that a neuron has a fixed voltage threshold for generating an action potential. However, experimental studies *in vivo* have suggested that the spike threshold is not constant but is highly variable (Azouz and Gray 2000; Henze and Buzsaki 2001; Chacron et al. 2007). Studies in the rodent hippocampus (Henze and Buzsaki 2001) and fish (Chacron et al. 2007) have demonstrated that the spike threshold increases after each action potential, which is referred to as “*threshold fatigue.”* We found that the spike threshold of the detailed neuron model jumps and decays exponentially after each spike with a time constant of ~100 [ms] (Fig. 4), suggesting that slow K^+^ currents may be the possible cellular mechanism underlying *threshold fatigue*. Other biophysical mechanisms, particularly Na^+^ currents, may underlie the threshold variability. The spike threshold also varies with the voltage derivative preceding a spike (Azouz and Gray 2000). Interestingly, it was shown that Na^+^ inactivation modulates the spike threshold, which varies with the membrane voltage with a small time constant (*τ*_*h*_(*v*) ≈ 2 ~ 10 [ms]) (Platkiewicz and Brette 2010; Fontaine et al. 2014) and Na^+^ inactivation can explain the voltage-dependence of the spike threshold observed in experiments (Platkiewicz and Brette 2011). The modulation of the spike threshold differs depending on its factor, in other words, the threshold modulation by slow K^+^ currents is slow and accumulative, whereas that by Na^+^ inactivation is rapid.

The instantaneous spike threshold of layer-5 pyramidal neurons has been estimated from the membrane potential recorded *in vitro* (Badel et al. 2008). The results suggest that the threshold modulation after a spike is described by the sum of two exponentials, ${\theta}_V\left(\mathrm{t}\right)\approx {\theta}_V^{\infty }+{A}_1{e}^{-\left(t-{t}_f\right)/{\tau}_1}+{A}_2{e}^{-\left(t-{t}_f\right)/{\tau}_2}$, where *θ*_*V*_(t) is the spike threshold and *t*_*f*_ is the most recent spike time. The fast components were *A*_1_ ≈ 10 [mV] and *τ*_1_ ≈ 20 [ms], whereas the slow components were *A*_2_ ≈ 1 [mV] and *τ*_2_ ≈ 100 [ms]. The detailed model used here reproduces the slow component in the threshold modulation; however, it does not reproduce the fast component. This is presumably due to the difference in Na^+^ current kinetics that describes the shape of an action potential. Indeed, it was reported that the spike waveform recorded from experiments is much shaper than that of Hodgkin–Huxley models (Badel et al. 2008).

### Functional implications of the slow K^+^ currents

It is well known that slow K^+^ currents induce the spike-frequency adaptation, which acts as a spike-triggered self-inhibition (Fig. 2; Benda and Herz 2003; Prescott and Sejnowski 2008). Several studies have proposed functional consequences of spike-frequency adaptation. For instance, the adaptation generates the “forward masking” effect, which suppresses the neuronal response under a prolonged stimulus (Liu and Wang 2001), improve signal transmission for low frequency stimulus (Chacron et al. 2007), and contributes to sparse and reliable coding (Farkhooi et al. 2013). Here, we have derived a simplified model that can reproduce the differential effects of slow K^+^ currents. The reduced model can accurately predict spike trains of the detailed neuron model (Fig. 7) and reproduce the *f*-*I* curve and spike train power spectrum (Data not shown).

Previous studies have suggested that slow K^+^ currents have differential effects on the coding property of a single neuron. For instance, *I*_M_ facilitates spike-timing coding because it improves the robustness of spike pattern against the input noise. In contrast, *I*_AHP_ enhances spike-rate coding, because it regularizes the spike train elicited by slow inputs (Prescott and Sejnowski 2008). It has also been suggested that *I*_M_ increases, whereas *I*_AHP_ decreases, the response to low-frequency input signals (Deemyad et al. 2012). Our analysis revealed a new differential effect underlying slow K^+^ currents (Fig. 8b, c), i.e., *I*_M_ suppresses firing irregularity regardless of the firing rate, whereas *I*_AHP_ suppresses the irregularity only at a low firing range (~3 [Hz]). This result suggests that neurons with *I*_AHP_ can contribute to the generation of rhythmical activity at a low firing rate. We hope that the reduced model will be useful for analyzing how the slow K^+^ currents impact on the coding properties of single neurons and neural populations.

## Appendix A: Approximate formulae for spike triggered ionic currents: ***η***_M_, ***η***_AHP_

We derive approximate formulae for the spike-triggered current induced by slow K^+^ currents. For simplicity, we consider a situation in which the neuron generates a spike at *t* = 0 [ms], and the input current is constant.

### A.1. M current

We replace a spike with a rectangular pulse with a peak voltage *v*_1_ and a width *w*_*sp*_ (Destexhe 1997). The differential equation for the *p* (Table 1) can be simplified to

37 $$ \frac{d\tilde{p}}{dt}=\left\{\begin{array}{c}\hfill -\frac{\tilde{p}-{p}_{\infty}\left({v}_1\right)}{\tau_p\left({v}_1\right)}\ \left(\mathrm{Spike}:0<t<{w}_{\mathrm{sp}}\right)\hfill \\ {}\hfill -\frac{\tilde{p}-{p}_{\infty}\left(\overline{v}\right)}{\tau_p\left(\overline{v}\right)}\ \left(\mathrm{Otherwise}:{w}_{\mathrm{sp}}<t\right),\ \hfill \end{array}\right. $$

where $\tilde{p}$ is an approximation of *p* and $\overline{v}$ is an equilibrium voltage after a spike. The solution of Eq. (37) is

38 $$ \tilde{p}(t)=\left\{\begin{array}{c}\hfill {p}_{\infty}\left({v}_1\right)+\left(\tilde{p}(0)-{p}_{\infty}\left({v}_1\right)\right)\ {e}^{-t/{\tau}_p\left({v}_1\right)}\kern1.25em \left(0<t<{w}_{\mathrm{sp}}\right)\hfill \\ {}\hfill\ {p}_{\infty}\left(\overline{v}\right)+\left(\tilde{p}\left({w}_{\mathrm{sp}}\right)-{p}_{\infty}\left(\overline{v}\right)\right)\ {e}^{-\left(t-{w}_{\mathrm{sp}}\right)/{\tau}_p\left(\overline{v}\right)}\ \left({w}_{\mathrm{sp}}<t\right).\ \hfill \end{array}\right. $$

Based on Eq. (38), the equilibrium conductance is given by ${\overline{g}}_{\mathrm{M}}={g}_{\mathrm{M}}\ {p}_{\infty}\left(\overline{v}\right).$ The spike-triggered current after the spike is written as

39 $$ {\eta}_{\mathrm{M}}(t)\approx {g}_{\mathrm{M}}\left(\tilde{p}(t)-{p}_{\infty}\left(\overline{v}\right)\right)\ \left(v(t)-{E}_K\right), $$

where *v*(*t*) is the membrane voltage. By substituting Eq. (38) into (39), we obtain

40 $$ {\eta}_{\mathrm{M}}(t)\approx {a}_{\mathrm{M}}{e}^{-t/{\tau}_p\left(\overline{v}\right)}, $$

where ${a}_{\mathrm{M}}={g}_{\mathrm{M}}\left(\overline{v}-{E}_K\right)\left(\tilde{p}\left({w}_{\mathrm{sp}}\right)-{p}_{\infty}\left(\overline{v}\right)\right)\ {e}^{w_{\mathrm{sp}}/{\tau}_p\left(\overline{v}\right)}$. A more accurate formula can be obtained by incorporating the spike waveform. Voltage after a spike can be approximated by an exponential function $v(t)\approx \delta v{e}^{-t/{\tau}_m}+\overline{v}$,

41 $$ {\eta}_{\mathrm{M}}(t)\approx {a}_M^1{e}^{-t/{\tau}_m}+{a}_M^2{e}^{-t/{\tau}_p\left(\overline{v}\right)}, $$

where *τ*_*m*_ is the membrane time constant and

$$ {a}_{\mathrm{M}}^1={g}_{\mathrm{M}}\delta v\left(\tilde{p}\left({w}_{\mathrm{sp}}\right)-{\tilde{p}}_{\infty}\left(\overline{v}\right)\right){e}^{w_{\mathrm{sp}}/{\tau}_p\left(\overline{v}\right)},\kern0.24em {a}_{\mathrm{M}}^2={a}_{\mathrm{M}}. $$

We assumed that the membrane time constant is much smaller than the time constants of p-gate: ${\tau}_m\ll {\tau}_p\left(\overline{v}\right)$. Equation (41) is more accurate than Eq. (40) in the short period (*t* < 30 [ms]) after a spike. In this study, we adopted the simpler formula (40) for simplicity.

### A.2. AHP current

Because the Ca^2+^ current is fast (Fig. 3a) compared to the time constant of Ca^2+^ outflux (: *τ*_Ca_ ≈ 200 [ms]), we can approximate the calcium current with a short pulse, *I*_Ca_ ≈ *q*_Ca_*δ*(*t*). The Ca^2+^ concentration after a spike at time *t* is

42 $$ \left[{\mathrm{Ca}}^{2+}\right]\approx \delta \mathrm{C}\mathrm{a}\ {e}^{-t/{\tau}_{\mathrm{Ca}}}+{\left[{\mathrm{Ca}}^{2+}\right]}_{\infty }, $$

where *δ*Ca = − 5.0 × 10^4^ × *q*_Ca_/F represents Ca^2+^ influx during a spike. Because the Ca^2+^ concentration is very small, s-gate time constant can be approximated as ${\tau}_s={\left({\alpha}_s+{\beta}_s\right)}^{-1}\approx {\beta}_s^{-1}=:{\tilde{\tau}}_s$. Hence, we obtain

43 $$ \frac{d\tilde{s}}{dt}=-\tilde{s}/{\tilde{\tau}}_s+0.01\left[{\mathrm{Ca}}^{2+}\right](t), $$

where $\tilde{s}$ is an approximation of the s-gate variable *s*. By substituting Eq. (42) into (43), we can solve the differential equation analytically as,

44 $$ \tilde{s}(t)={a}_s\left({e}^{-t/{\tau}_{\mathrm{Ca}}}-{e}^{-t/{\tilde{\tau}}_s}\right)+{s}_{\infty }, $$

where ${a}_s=\frac{0.01{\tau}_{\mathrm{Ca}}{\tilde{\tau}}_s}{\tau_{\mathrm{Ca}}-{\tilde{\tau}}_s}\delta \mathrm{C}\mathrm{a}$, ${s}_{\infty }=0.01{\left[{\mathrm{Ca}}^{2+}\right]}_{\infty }{\tilde{\tau}}_s$. As with the case of *I*_M_, the equilibrium conductance is given by ${\overline{g}}_{\mathrm{AHP}}={g}_{\mathrm{AHP}}{s}_{\infty }$. The spike-triggered current is

45 $$ {\eta}_{\mathrm{AHP}}(t)\approx {g}_{\mathrm{AHP}}\left(\tilde{s}(t)-{s}_{\infty}\right)\left(v(t)-{E}_{\mathrm{K}}\right), $$

By substituting Eq. (44) into (45) and replacing the voltage with its equilibrium value $\overline{v}$, we obtain

46 $$ {\eta}_{\mathrm{AHP}}(t)\approx {a}_{\mathrm{AHP}}\left({e}^{-t/{\tau}_{\mathrm{Ca}}}-{e}^{-t/{\tilde{\tau}}_s}\right), $$

where $$ {a}_{\mathrm{AHP}}={g}_{\mathrm{AHP}}\left(\overline{v}-{E}_{\mathrm{K}}\right){a}_s. $$

## Appendix B: Approximate formula for spike threshold: ***h***_M_(***t***), ***h***_AHP_(***t***)

We derive an approximate formula that describes how the slow K^+^ currents modulates spike threshold. Close to spike threshold, the membrane voltage of a neuron can be described by the exponential integrate and fire model (Fourcaud-Trocmé et al. 2003; Platkiewicz and Brette 2010),

47 $$ {C}_m\frac{dV}{dt}=F(V)=-{g}_{\mathrm{tot}}\left(V-{E}_{\mathrm{tot}}\right)+{g}_{\mathrm{tot}}{\Delta}_T{e}^{\left(V-{V}_T\right)/{\Delta}_T}-{I}_{\mathrm{adp}}(t), $$

where ${I}_{\mathrm{adp}}(t)={\displaystyle {\sum}_{k:\ {t}_k<t}{\eta}_{\mathrm{ion}}\left(t-{t}_k\right)}$ is the spike-triggered current induced by the slow K^+^ currents. The spike threshold *θ*_*V*_ defined by a critical voltage above which the neuron emits a spike, *F*(*θ*_*V*_) = 0, is given by

48 $$ {\theta}_V\approx {V}_T+{\Delta}_T \log \left(\frac{V_T-{E}_{\mathrm{tot}}+R{I}_{\mathrm{adp}}}{\Delta_T}\right), $$

where *R* = *g*_tot_^− 1^ is the membrane resistance. If *RI*_adp_ is small compared to *V*_*T*_ − *E*_tot_, Eq. (48) can be simplified further,

49 $$ {\theta}_V\approx {V}_T+{\Delta}_T \log \left(\frac{V_T-{E}_{\mathrm{tot}}}{\Delta_T}\right)+\frac{R{\Delta}_T}{V_T-{E}_{\mathrm{tot}}}{I}_{\mathrm{adp}}. $$

The variation of spike threshold by an ionic current can be given by

50 $$ {h}_{\mathrm{ion}}\approx \frac{R{\Delta}_T}{V_T-{E}_{\mathrm{tot}}}{\eta}_{\mathrm{ion}}. $$

We can see from Eq. (50) that the threshold variation *h*_ion_ is approximately proportional to the spike-triggered current *η*_ion_.

## Appendix C: Relating the reduced model to the MAT model

By substituting Eqs. (19) − (22) into (17), the effective spike threshold modulated by a spike can be written as

51 $$ \begin{array}{l}H(t)=-\delta V{e}^{-\left(t-{w}_{\mathrm{sp}}\right)/{\tau}_{\mathrm{m}}}+\frac{a_{\mathrm{M}}}{C_m}{f}_{De}\left({\tau}_p\left(\overline{v}\right),{\tau}_m\right)+\frac{a_{\mathrm{AHP}}}{C_m}\left\{{f}_{De}\left({\tau}_{\mathrm{Ca}},{\tau}_m\right)-{f}_{De}\left({\tilde{\tau}}_s,{\tau}_m\right)\right\}\\ {}\kern3em +{b}_{\mathrm{M}}{e}^{-t/{\tau}_p\left(\overline{v}\right)}+{b}_{\mathrm{AHP}}\left({e}^{-t/{\tau}_{\mathrm{Ca}}}-{e}^{-t/{\tilde{\tau}}_s}\right),\ \end{array} $$

where ${f}_{De}\left({\tau}_1,{\tau}_2\right):=\left({e}^{-t/{\tau}_1}-{e}^{-t/{\tau}_2}\right)/\left({\tau}_2^{-1}-{\tau}_1^{-1}\right)$ and the time constants are given in Eqs. (19) and (20). If we assume that the membrane time constant is much smaller than the slow K^+^ time constants: ${\tau}_m\ll {\tau}_p\left(\overline{v}\right),\ {\tau}_{\mathrm{Ca}},\ {\tilde{\tau}}_s$, the formula can be simplified further,

52 $$ H(t)\approx {\alpha}_0{e}^{-t/{\tau}_m}+{\alpha}_{\mathrm{M}}{e}^{-t/{\tau}_p\left(\overline{v}\right)}+{\alpha}_{\mathrm{AHP}}\left({e}^{-t/{\tau}_{\mathrm{Ca}}}-{e}^{-t/{\tilde{\tau}}_s}\right), $$

where the weights are ${\alpha}_0=-\delta V{e}^{w_{\mathrm{sp}}/{\tau}_{\mathrm{m}}}-{a}_M/{g}_{\mathrm{tot}}$, *α*_M_ = *a*_*M*_/*g*_tot_ + *b*_M_, *α*_AHP_ = *a*_AHP_/*g*_tot_ + *b*_AHP_. The slow weights can be related to the slow K^+^ parameters by using Eqs. (40), (46), (50),

53 $$ \begin{array}{cc}\hfill {\alpha}_{\mathrm{M}}\propto {a}_{\mathrm{M}} \propto {g}_{\mathrm{M}}\left(\overline{v}-{E}_K\right)\delta p,\hfill & \hfill {\alpha}_{\mathrm{AHP}}\propto {a}_{\mathrm{AHP}} \propto {g}_{\mathrm{AHP}}\left(\overline{v}-{E}_{\mathrm{K}}\right)\delta \mathrm{C}\mathrm{a}\hfill \end{array}, $$

where $\delta p:=\tilde{p}\left({w}_{\mathrm{sp}}\right)-{p}_{\infty}\left(\overline{v}\right)$ and *δ*Ca are the changes in *p* and [Ca^2+^] during a spike.
